# Super-Resolution Image Optimisation Based on Gradient Iterative Fast Diffraction-Free Spot Algorithm

**DOI:** 10.3390/s25103221

**Published:** 2025-05-20

**Authors:** Chen Yu, Ying Liu, Linhan Li, Guangpeng Zhou, Boshi Dang, Jie Du, Junlin Ma, Site Zhang

**Affiliations:** 1 Changchun Institute of Optics, Fine Mechanics and Physics, Chinese Academy of Sciences, Changchun 130033, China; yuchen21@mails.ucas.ac.cn (C.Y.); lilinhan22@mails.ucas.ac.cn (L.L.); zhouguangpeng19@mails.ucas.ac.cn (G.Z.); dangboshi@163.com (B.D.); dj_ciomp@163.com (J.D.); michael8446@126.com (J.M.); zhangsite@ciomp.ac.cn (S.Z.); 2 University of Chinese Academy of Sciences, Beijing 100049, China

**Keywords:** elimination of diffraction spots, microscopy, fast autofocus, computer vision

## Abstract

Diffraction significantly deteriorates the quality of the laser image, causing severe degradation that undermines the theoretical performance parameters of the autofocus system. In this paper, we conduct a comprehensive analysis of the non-uniform features of the images. To enhance the imaging quality of each individual image, we propose a de-diffraction algorithm based on gradient iteration. This algorithm is capable of rapidly removing the interference spots resulting from diffraction and restoring the distorted laser spots. By doing so, it effectively eliminates the inevitable reduction in the autofocus resolution and focusing accuracy caused by diffraction. Furthermore, the proposed calculation model for the intra-localisation interval significantly improves the convergence of the iterative calculation process. Through experiments, it has been verified that, under the same conditions, the interlayer resolution between the reflective surfaces of the samples processed using this algorithm is increased to a quarter of the original value. This remarkable improvement in resolution, which far exceeds the microscope’s inherent resolution, demonstrates that the algorithm successfully achieves super-resolution for the microscope.

## 1. Introduction

Autofocus represents a crucial application within the realm of optical microscopes, and the quality of its imaging serves as a pivotal indicator for evaluating the overall performance of the microscope [1,2,3,4,5]. In industrial settings, certain inevitable circumstances, including an uneven light source and the presence of noise interference, can give rise to a series of issues. These issues encompass a low imaging resolution and a suboptimal signal-to-noise ratio of the microscope [6,7,8]. Such problems not only impede the practical observation of samples but also significantly diminish the microscope’s original accuracy specifications. Consequently, in the context of a specific experimental environment, the post-processing of images takes on a particularly vital significance. With the swift advancement in digital technologies like the Internet of Things, the value of digital images has been soaring exponentially.

The enhancement in image processing holds paramount importance in the construction of optical systems. It can effectively compensate for deficiencies in image quality stemming from hardware limitations and other factors. By doing so, it significantly mitigates errors caused by subpar imaging quality, reducing inaccuracies inherent to the optical instruments themselves and, thereby, enhancing overall precision. Remarkably, this improvement is achieved without the need to increase the number of lenses, enlarge the aperture, or undertake other hardware modifications, simultaneously alleviating the complexities associated with installation and adjustment. For instance, through image processing, tasks such as aberration correction and chromatic aberration elimination become feasible. These capabilities enable the optimisation of the optical imaging system, directly contributing to a notable enhancement in imaging quality. In particular, when dealing with incomplete or missing image information, image enhancement techniques can be employed to reconstruct high-quality models, facilitating more efficient optical design processes. Owing to the inherent characteristics of optical image processing systems, a high level of automation can be achieved. This automation allows the optical system to operate in a non-contact manner, with high precision, at a rapid pace, and, to a certain extent, fully autonomously, thus revolutionising the field of optical system construction and operation.

When it comes to diverse images, their processing approaches can draw upon either traditional methodology or deep learning [9,10,11,12,13,14,15]. Traditional methods typically hinge on image feature representations, in which the features are devised by humans. These methods are capable of processing images of varying dimensions within a relatively short timeframe. They are somewhat subjective; yet, at the same time, they are simple and efficient [16,17]. For images within different scopes, the processing is rather flexible. Grounded in the function algorithm of image grayscale transformation, diverse mathematical function calculations can bring about different alterations to the pixel grayscale values. Nonetheless, as this approach places a greater reliance on hand-crafted features, it lacks the ability to extract high-level features of the image. With a relatively low upper limit in feature extraction, it consequently results in a high error rate in fields such as image recognition [18]. In sharp contrast, deep learning endows the outcomes with greater reliability by capitalising on training conducted on extensive datasets. Through the process of fitting features and classifiers into network training and leveraging error backpropagation for learning and iterative refinement, it demonstrates a notably higher accuracy rate in domains that involve large datasets, such as image extraction and image recognition [19,20,21,22]. Nevertheless, the generation of results in deep learning hinges on substantial data support. Additionally, it demands a high degree of variability in the features within the dataset across different classifications. As a result, when there is a lack of regular variation in the image subject matter and when there is an overlap of feature contents, it proves challenging for deep learning to achieve the superior recognition of the target [23,24].

Optical imaging inevitably encounters the adverse impacts stemming from diffraction. Diffraction produces a large number of stray spots that are randomly dispersed around the imaging spot and intensify as the light intensity of the main spot increases. Moreover, the diffracted spots that cling between the laser spots undermine the microscope’s super-resolution capability. Furthermore, when the laser spot is near the focal position of the objective lens, the light intensity of the diffracted spot is comparable to that of the main spot when the system is out of focus. This renders it impossible to distinguish between the diffracted spot and the main spot using the traditional intensity threshold method, thereby imposing specific demands on the image processing algorithm. On the other hand, due to the Gaussian propagation of the laser, the distribution of the light intensity of the laser spot captured by the detector is severely uneven. The center of the spot is brighter, while the upper and lower ends are dimmer. This unevenness is likely to give rise to singularities, which, in turn, weakens the focusing accuracy of the autofocus system and may lead to incorrect focusing.

In traditional image processing algorithms, the wavelet operation, filtering operation, and the like are among the commonly employed algorithms for denoising purposes [25]. Nevertheless, the diffracted spot exhibits a broad distribution pattern, and there is no distinct threshold disparity between the diffracted spot and the laser spot within the frequency domain. Consequently, the denoising procedure merely diminishes the high-frequency features of the image and falls short of eradicating the diffraction phenomenon. When traditional detection operators, such as the Sobel operator, are utilised for extracting the edges of the target, the gradient variation in the pixel points at the edge of the diffracted spot closely resembles that of the laser spot. This situation leads to an inadequate elimination of diffraction when the threshold is set low. When the threshold is increased, not only is the elimination of diffraction still insufficient, but it also significantly reduces the main body information of the image.

Taking into account the random arrangement of the diffraction effect, the adhesion between the main spot and the diffraction noise, as well as the necessity to capture the special details of the spot, this paper conducts research and designs a novel algorithm. Building upon traditional algorithms, this new approach aims to eliminate diffracted spots and restore image quality. It enhances the accuracy of recognising the diffraction effect and compensates for the deficiencies in image quality caused by hardware limitations. By leveraging the gradual change in the gray values of pixels in the horizontal direction of the spot, the image is partitioned. Singularities within the intervals are regarded as noise and removed, thereby enhancing the interlayer resolution of the microscope. To prevent redundant iterative operations, the image undergoes subject enhancement, and background pixel points that do not carry useful information are excluded from the calculation process. Compared with traditional operators, the algorithm proposed in this paper circumvents the errors arising from the multi-directional weight calculation of gray values, reducing the error rate in interpreting diffracted spots and main spots. Additionally, for the “broken points” and “dark pixel points” generated due to the uneven distribution of the half-ring spot, this algorithm is capable of identifying and differentiating them from the image background. This is carried out even with the aim of improving the image quality to enhance the signal-to-noise ratio of the image, significantly boosting the accuracy of the focusing system. Experimental verification has shown that, under this algorithm, the limiting resolution distance between the reflective surfaces of the samples can be increased to a quarter of the original value.

## 2. Principle of Autofocus System

**Figure 1:**
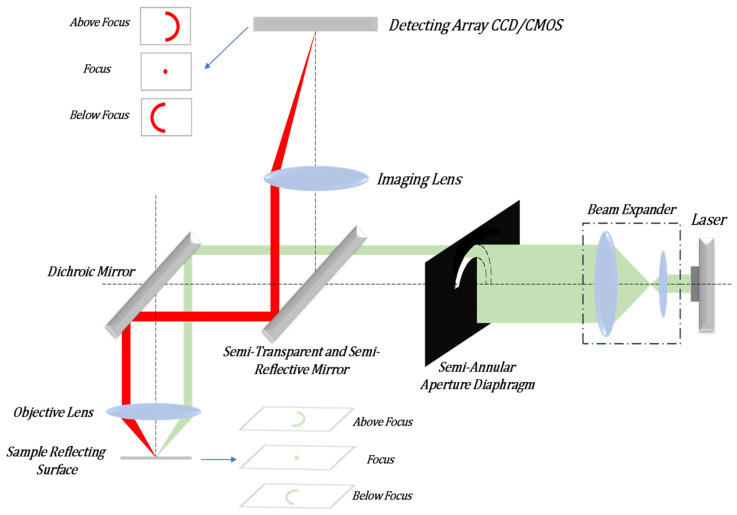
Schematic diagram of the autofocus system for imaging multiple reflective surfaces of a sample.

In order to achieve a multifaceted observation of biological samples, we propose a novel autofocus system. The system can image multiple reflective surfaces of a transparent sample and can determine the out-of-focus size and direction of each reflective surface based on the shape and size of the spot on the detector. The semi-annular laser Autofocus (AF) microscope is a remarkable instrument that enables the observation of multiple layers within transparent samples. It outperforms other active autofocus microscopes in terms of resolution and focusing accuracy. The autofocus system of this microscope boasts an impressive theoretical resolving power: 0.68 μm in the horizontal direction and 0.60 μm along the optical axis. This outstanding performance allows it to attain the super-resolution ability that surpasses the capabilities of traditional optical microscopes. The schematic illustration is presented in Figure 1. As depicted in Figure 1, the laser emits infrared laser light with a wavelength of 785 nm. This emitted light undergoes collimation and expansion processes. Subsequently, it is modulated into semi-annular parallel laser light via a semi-annular aperture diaphragm. The light then proceeds to enter the microscope system through a beam splitter. Inside the system, it is reflected by a dichroic mirror into the microscope objective lens and, ultimately, reaches the transparent sample. Owing to variations in the degree of being out-of-focus, the distinct reflective surfaces of the sample will give rise to semi-annular spots that differ in both radius and orientation. Due to the reflection from the sample, the light beam is redirected back into the autofocus system. Finally, the light is converged by a lens and forms a magnified image on the Charge-Coupled Device (CCD). By observing the length of the radius and the orientation of the semi-annular image on the CCD, one can determine both the direction of the out-of-focus condition of the sample’s reflective surface and the corresponding out-of-focus distance. In the schematic diagram of Figure 1, it is assumed by default that the current position of the middle reflective surface of the sample coincides with the focal position of the microscope objective lens.

**Figure 2:**
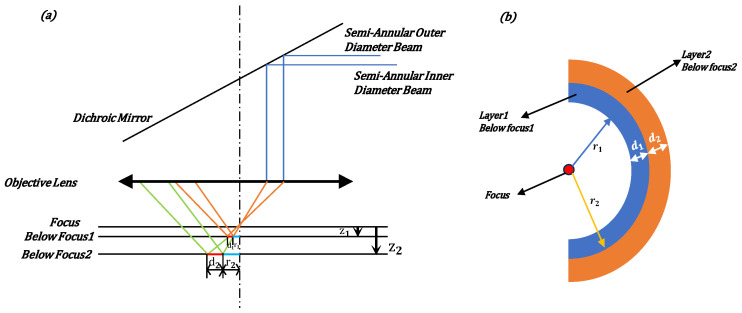
(**a**) depicts the trajectory of the incident laser light on various reflective surfaces when the reflective surface of the sample is in an out-of-focus state. (**b**) shows the image formed by the laser light on the reflective surface of the sample.

When the sample’s reflective surface is precisely at the focal point, the laser light converges onto that particular reflective surface and manifests as a point. Conversely, when the sample’s reflective surface is out of focus, the laser light on the reflective surface forms an image in the shape of a semi-annular spot. Moreover, depending on whether the amount of being out-of-focus is positive or negative, the orientation of the semi-annular spot will vary. The underlying principle is illustrated in Figure 2. In this context, $z_{1}$ represents the positive out-of-focus distance of the first level of the sample, while $z_{2}$ denotes the positive out-of-focus distance of the second level of the sample. $r_{1}$ is the length from the center of the optical axis to the inner diameter of the semi-annular spot formed by the laser light when imaging on the first level of the sample, and $d_{1}$ is the difference in length between the inner and outer diameters of the semi-annular spot, which indicates the width of the ring. Similarly, $r_{2}$ is the length from the center of the optical axis to the inner diameter of the semi-annular spot formed by the laser light when imaging on the second level of the sample, and $d_{2}$ represents the width of the semi-annular.

As is evident from Figure 2, the distances between the inner and outer diameters of the laser spots that are formed on the reflective surfaces with varying defocusing distances are not the same. Moreover, there exists a certain relationship between the width of the ring (that is, the difference between the inner and outer diameters of the semi-annular) and the defocusing distance of the reflective surface, which is as follows:

(1) $$\frac{d_{1}}{d_{2}}=\frac{z_{1}}{z_{2}}$$

And the relationship between the distance from the inner diameter of the laser spot to the center origin of each reflecting surface is as follows:

(2) $$\frac{r_{1}}{r_{2}}=\frac{z_{1}}{z_{2}}$$

Therefore, the out-of-focus distance of the reflecting surface is directly proportional to the size of the inner diameter of the semi-annular spot and the ring width of its corresponding image.

When two reflective surfaces within a transparent sample are extremely close to one another, as illustrated by the convergence of $z_{1}$ towards $z_{2}$, it gives rise to a scenario where the spots projected onto the CCD overlap. In other words, $d_{1}$ and $d_{2}$ coincide, rendering it impossible for the autofocus system to differentiate between these two reflective surfaces. To ensure accurate focusing, there must be a specific distance maintained between the sample’s reflective surfaces. The distance between two reflective layers, corresponding to the situation where the semi-annular spot on the CCD and the adjacent spot are just separated, is referred to as the interlayer resolution. In terms of the performance on the CCD, when the two semi-annular spots are just overlapping, the distance between the inner diameters of the two semi-annular shapes is precisely $d_{1}$. Mathematically, this is expressed as $r_{2}$ = $r_{1}$ + $d_{1}$.

**Figure 3:**
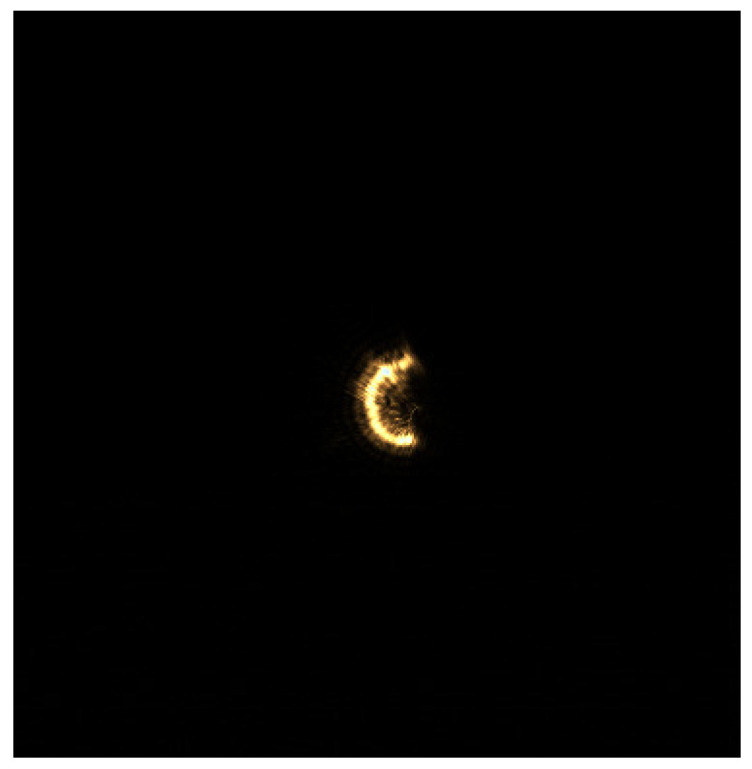
Laser imaging of a single reflective surface of the sample.

When multiple reflective surfaces are in an out-of-focus state, the accuracy of the autofocus system hinges upon the interlayer resolution. The magnitude of the interlayer resolution, in turn, is influenced by the imaging quality of the CCD. During the experiment, the CCD displays multiple enlarged and nested semi-annular spots. Even when the distance between the reflective surfaces meets the theoretical interlayer resolution requirement, the diffraction effect produced by the semi-annular aperture diaphragm leads to the appearance of diffracted spots between the main spots on the CCD. Consequently, in order to prevent the two spots from overlapping, it is necessary that we increase the distance between the reflective surfaces beyond the interlayer resolution distance. This additional increase in distance weakens the resolving power of the microscope. Simultaneously, due to the non-uniform propagation of the laser, the intensity of the spots imaged on the CCD is uneven. As a result, some of the spot pixels are easily mistaken for the background. This confusion prevents the focusing system from accurately feeding back the degree of being out-of-focus, and, thus, the system fails to achieve the theoretical focusing accuracy. When there is merely a single reflective surface, the experimental imaging image is presented in Figure 3. It can be observed from the figure that the diffracted spots are closely arranged on the outer side of the semi-annular spot, thereby expanding the original pixel area. When the multiple reflective surfaces of the sample are imaged simultaneously, the diffracted spots overlap with one another, severely deteriorating the image quality. To address the aforementioned issues, this paper employs a gradient iterative algorithm to offset the impact of diffraction caused by hardware limitations. This algorithm aims to improve the imaging quality and enhance the performance of the system by effectively dealing with the diffraction-related problems and reducing their negative effects on the captured images.

## 3. Algorithmic Model

In order to successfully extract the image target and remove the diffracted spots, the iterative gradient detection algorithm is utilised here for extracting the main subject light spot. Typically, in an image, the area where the grayscale undergoes a drastic change represents the boundary line. Thus, the starting edge of the light spot corresponds to the positive and negative positions where there is a significant gradient change. Although the gradient change in the edge grayscale of the diffracted light spot also adheres to the jumping rule, when compared to the semi-annular light spot, the change in the grayscale value of the central pixel is more frequent. Regarding the semi-annular spot, the lateral distribution of light intensity is more uniform, and the gradient difference tends to approach 0, indicating a strong correlation. When moving from the starting point towards the end point for each row of pixels in the image, those adjacent and closely packed pixel points with a gradient nearly equal to zero, located between the drastic positive and negative jumps in the grayscale value, are the imaging points of the semi-annular spot. On the other hand, the pixel points between each semi-annular imaging point can be considered as the background of the image. In contrast, the pixel grayscale values of the diffractive spots change in a chaotic and irregular manner. As a result, the number of pixel points with a zero-value gradient situated between the edge pixel points is significantly smaller compared to that of the semi-annular spot. This serves as a key criterion for differentiating between the two. Finally, through the iterative calculation of each row of pixels in the image, the calibration of the pixel points for each row of the entire image is accomplished. This process enables a more accurate separation and identification of the semi-annular spot pixels from the diffractive spot pixels and the background pixels, thereby enhancing the overall quality and clarity of the image analysis. It effectively leverages the differences in gradient characteristics to achieve the goal of extracting the target image and eliminating the interference of diffractive spots.

Before the image data are processed, the gray value of each pixel point in the image is normalised from the initial range of 0–255 to 0–1. Let the grey value of a particular pixel point be $g_{k}$; the center difference of the pixel point is defined as follows:

(3) $$∆g_{k}=\frac{g_{k+1}-g_{k-1}}{2}$$

For the image f(x), its gradient value per row of pixel iterations is the convolution of the transverse difference matrix with the image, i.e.,

(4) $${∆G}_{x}=\left[1\;0-1\right]*f\left(x\right)$$

**Figure 4:**
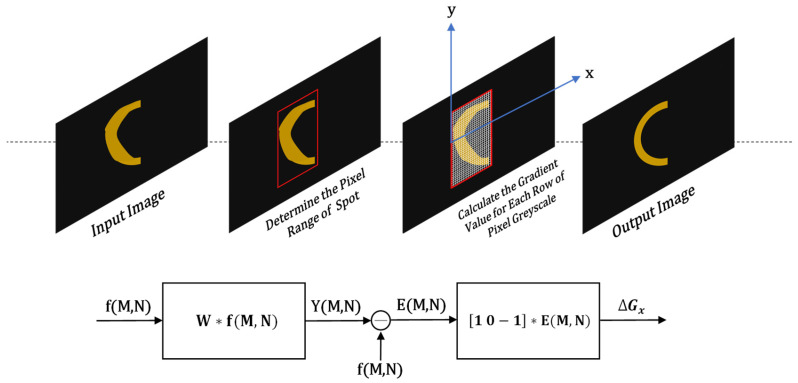
Schematic flow of the diffraction spot removal algorithm.

The value of $∆g_{k}$ serves as an indicator of the intensity of the gradient change in the horizontal pixel light intensity. When moving from the starting point to the end point of the pixel points, at the outer diameter edge of the semi-annular spot, the light intensity undergoes a transition from weak to strong, which is reflected by $∆g_{k}$ > 0. On the other hand, at the inner diameter edge of the semi-annular spot, the light intensity changes from strong to weak, corresponding to $∆g_{k}$ < 0. For the pixel points located between the inner and outer diameters of the semi-annular spot, the value of $∆g_{k}$ is approximately 0. In contrast, for the pixel points of the diffractive spots, the variation of $∆g_{k}$ shows more frequent positive and negative fluctuations. Moreover, the number of pixel points that satisfy $∆g_{k}$ ≈ 0 within the intervals where $∆g_{k}$ > 0 and $∆g_{k}$ < 0 is relatively small. That is to say, the gradient of the diffracted light fluctuates more frequently. By taking this characteristic as a criterion, these diffractive spot pixel points can be identified and eliminated. The flowchart of the algorithm is shown in Figure 4, which removes the diffractive spots from the input image and fits the desired output image based on the disordered nature of the diffractive spot light intensity variations described above.

When the proportion of background pixel points in an image is significantly high, performing calculations on every single row of pixels is unnecessary. As illustrated in Figure 4, in order to guarantee rapid image processing, prior to each iterative operation, the approximate pixel interval encompassing the light spot is detected. By confining the operations within this specific interval, there is a significant reduction in the image processing time. The background pixel points exhibit a high degree of correlation. In contrast, the light spot possesses distinct characteristics that set it apart from the surrounding background pixel points. This distinction allows for the determination of the pixel location of the light spot. It is achieved by calculating the disparity between the actual grayscale value of a pixel point and the predicted grayscale value of the pixel point. Through this comparison, the algorithm can effectively identify the region where the light spot is situated. This targeted approach enables the algorithm to focus its computational resources on the relevant area of the image rather than processing the entire image. Its arithmetic model is as follows:

(5) Y(m,n)=∑∑W(l,k)f(m−l,n−k)

where *m* = 0, …, *M* − 1; *n* = 0, …, *N* − 1; *l*, *k* ∈ $S_{j}$. *f* is the M × N input image, *Y* is the predicted background image, *W* is the weight, and $S_{j}$ is the set of background pixel points selected for the computation. Thus, the difference between the input image and the predicted background image can be expressed as follows:

(6) E(m,n)=f(m,n)−Y(m,n)

**Figure 5:**
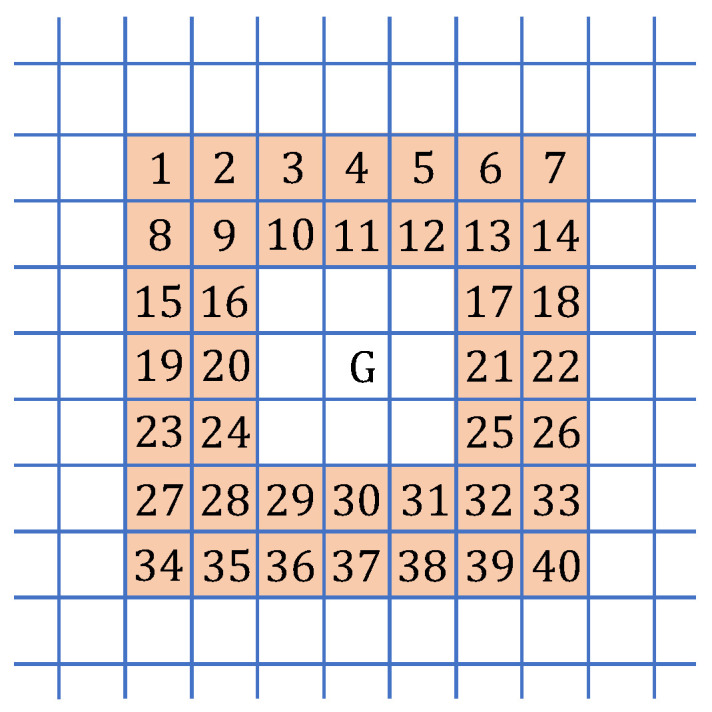
Schematic of the $S_{j}$ pixel point set.

When the difference *E* exceeds the set threshold, the pixel point can be considered a target pixel point, not a background pixel point. When computing the predicted background grayscale value of pixel *G*, the selection of the set $S_{j}$ of background pixels, which consists of 40 pixels located outside the 3 × 3 neighbourhood of the pixel *G*, is an important step in the algorithm. The schematic is shown in Figure 5:

In this paper, *W* = 1/40, the average value of the selected background grey scale is used as a reference to calculate the predicted grey scale value of point *G*. When the difference E corresponding to point *G* is larger than the threshold, point *G* is considered to be the pixel point of the light spot, and, here, there is no distinction between a diffracted light spot or a target light spot that wants to be extracted. The edge position of the pixel point of the spot is taken as the interval of the spot distribution, and the iterative operation of the gradient is performed in the interval to extract the target spot. At the same time, the autofocus system determines how many reflective surfaces are imaged based on the number and distribution of intervals.

**Figure 6:**
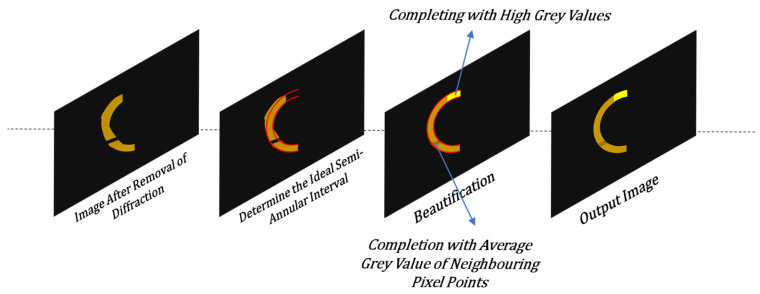
Schematic of the beautification of the imaged spot after de-diffraction.

When the quality of the semi-annular spot in the input image is subpar and the resulting imaging remains unsatisfactory even after the de-diffraction process, additional refinement is necessary. The pixel width at the horizontal center of the spot serves as a crucial reference point. By using this measurement, one can determine the ideal dimensions for the inner and outer diameters of the semi-annular shape. This step helps in standardising the spot’s appearance and making it more suitable for further analysis. For the pixel points in the image, those that lie outside the semi-annular interval are straightforwardly assigned the background gray value. This effectively removes any extraneous or interfering elements that are not part of the relevant spot region. The pixel points situated between the inner and outer diameters require a more nuanced approach. When the gray value of a pixel point within this interval is too low, it is replaced with the gray value of the adjacent longitudinal pixel point as a reference. This is carried out in an attempt to enhance the contrast and make the spot more distinguishable. However, if the newly replaced gray value is still too low and makes it difficult to differentiate the pixel from the background, a more drastic measure is taken, and the pixel is assigned a gray value of 1. This ensures that the spot stands out clearly against the background, improving the overall quality of the image. The schematic diagram of the algorithm is shown in Figure 6.

**Figure 7:**
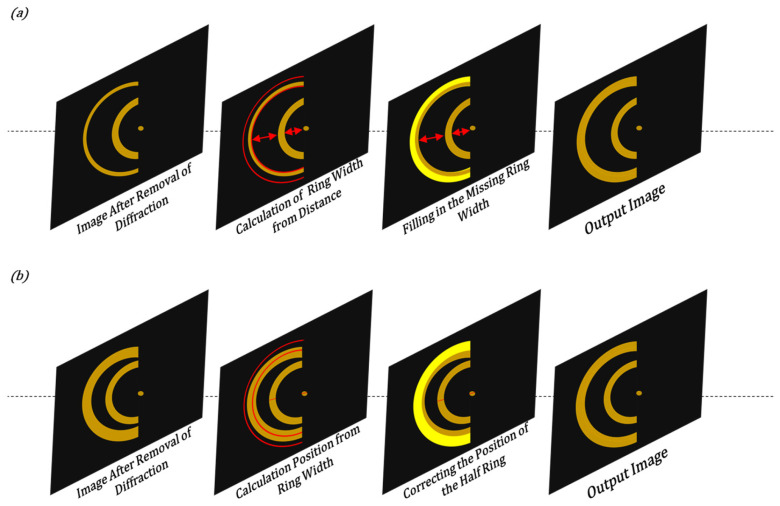
Schematic for correcting erroneous imaging using physical relations of rings. (**a**) is a schematic illustration of the incorrect ring width corrected according to the distance between the half rings. (**b**) is a schematic diagram of the use of ring width to adjust the position of the ideal semi-annular spot.

When dealing with an input image that contains multiple semi-annulars, the utilisation of Equations (1) and (2) based on the position of the center coordinates of each semi-annular spot allows for the determination of the ideal ring width size of each semi-annular spot and the degree of being out-of-focus of the reflective surface. Conversely, when the out-of-focus distance of each reflective surface of the sample is already known, an inverse calculation can be employed to obtain the corresponding center coordinates and ideal ring width of each semi-annular spot. This bidirectional relationship between the physical properties of the sample (out-of-focus distance) and the visual characteristics of the image (center coordinates and ring width) offers flexibility in both analysing the image data and predicting the expected image appearance based on prior knowledge of the sample. Therefore, if there are errors in the ring information in the input image after de-diffraction due to improper hardware mounting, including but not limited to the disappearance of a semi-annular spot, a missing part of a semi-annular spot due to the insufficient imaging of the semi-annular spot, a semi-annular spot at the wrong imaging position, etc., the wrongly imaged semi-annular spot can be corrected based on the information of the adjacent semi-annular spots in the image. As shown in Figure 7, the wrong ring width can be corrected based on the distance between the half rings. Or use the ring width to adjust the ideal semi-annular spot position.

## 4. Experimentation

**Figure 8:**
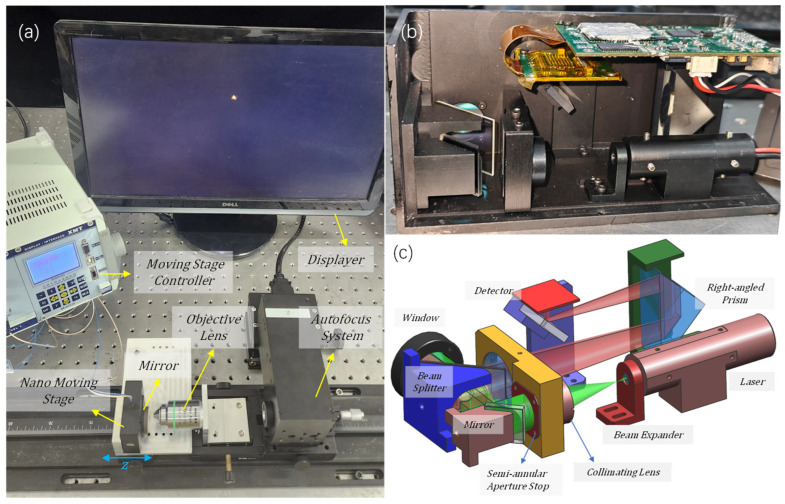
Schematic diagram of the experimental setup. (**a**) is the overall setup diagram of the experiment. (**b**) is the interior view of the AF system device. (**c**) is a simulation diagram of the parts of the AF system device.

In order to accurately obtain the sample spot image captured by the CCD, a hardware test platform was constructed, as shown in Figure 8a, which consists of an accuracy 1 nm nano-displacement stage, a planar reflector (Sunny Optical Technology Company Limited, Yuyao, China), used to simulate the sample reflective surface), a Nikon Plan Apo 20 X/0.75 objective lens (Nikon Corporation, Tokyo Metropolis, Japan), a semi-annular focusing system, and an image acquisition system. Figure 8b,c show the internal optical path of the focusing system. The light emitted from the laser passes through the beam expander mirror. It is expanded to match the size of the microscope’s pupil, which is 20 mm. Then, it goes through a semi-annular diaphragm plate with inner and outer diameters of 8 mm and 10 mm, respectively, generating the required semi-annular light beam. The semi-annular light beam is reflected by a reflector to a beam splitter mirror. Subsequently, it passes through a window and the microscope objective lenses before reaching the planar reflector. The reflected light travels back through the microscope objective lens and the window to the beam splitter. The light beam, upon being reflected by the beam splitter, enters the imaging mirror. Given that the focal length of the imaging mirror is as long as 200 mm, to prevent the system from being overly elongated, a right-angle prism is selected to effectively fold the optical path. Subsequently, a small reflector redirects the imaging beam back towards the detector, which is the CMV2000 model (CoreMorrow Ltd., Harbin, China). The image size of each element in this detector is 5.5 μm. By closely observing the shape of the focusing spot displayed on the screen (it is a principle that, the higher the symmetry of the focusing spot, the better the coaxiality), the coaxial adjustment of the focusing system, the microscope objective lens, and the planar reflector can be successfully achieved. By moving the displacement stage, a single-level imaging spot can be obtained. When located in the focal plane of the objective lens, a point is imaged on the CCD, and, when out of focus, a semi-annular spot is displayed.

**Figure 9:**
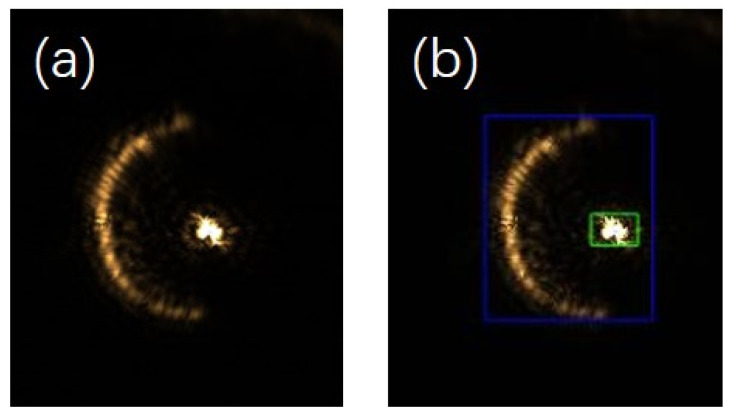
Imaging spot map captured by the CCD. (**a**) is the original unprocessed image. (**b**) is a picture of the spot interval detected by the algorithm.

When replacing the mirror with a biological sample that has multiple layers, multiple spots are imaged on the CCD. When the diffraction interference is weak, the picture captured by the CCD is shown in Figure 9a. The autofocus system recognises the spots in Figure 9a, determines the number of semi-annular spots and the approximate pixel intervals of the spots, and divides them according to the reflective surface of the sample, as shown in Figure 9b. As can be seen in Figure 9b, the autofocus system determines that there are currently two reflective surface spots and uses blue and green boxes to demarcate the detected target pixel point intervals.

**Figure 10:**
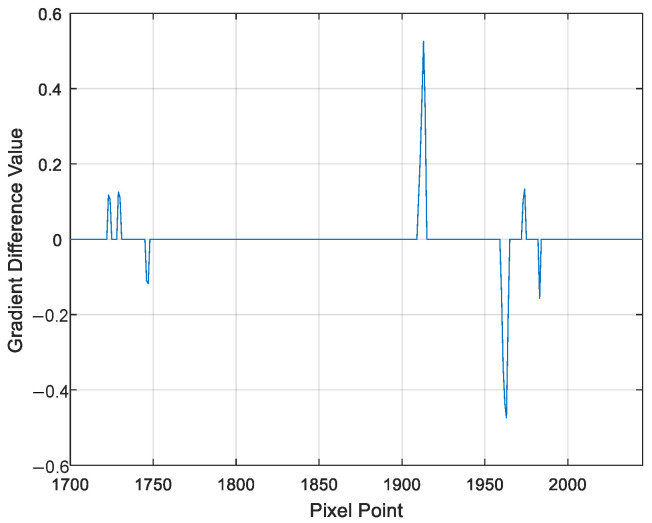
Schematic of the gradient change in the grey value of a pixel point in the same horizontal orientation.

Let us take a row of pixel points in the horizontal direction of the image as an illustration. When the horizontal pixel row is fixed at the 939th row, the position where the spot is imaged (the area indicated by the blue box in Figure 9b) is concentrated approximately from the 1700th column to the 2045th column, extending from left to right. Among this range, the focusing spot (the region marked by the green box in Figure 9b) is concentrated roughly from the 1885th column to the 2000th column. By performing a lateral difference operation on these pixel points, the gradient difference of each individual pixel point can be derived, as clearly shown in Figure 10.

**Figure 11:**
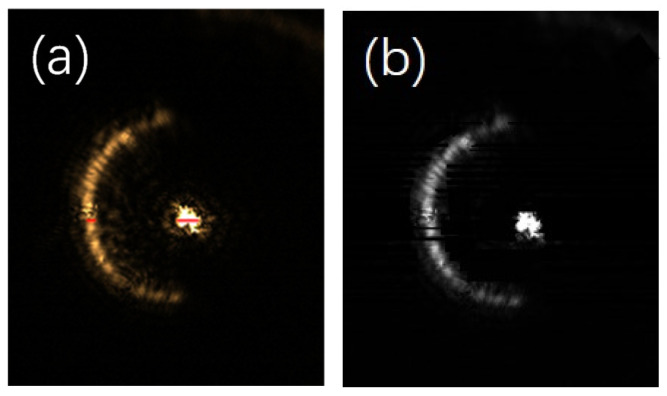
(**a**) is the set of pixel points (positions marked in red) corresponding to column pixels 1730–1745 and 1915–1961. (**b**) is the image after de-diffraction.

As is evident from Figure 10, the column pixel points for which the difference is zero are within the ranges of 1730–1745, 1915–1961, and 1976–1980. All three of these ranges exhibit the characteristic pattern of light intensity changing from dark to light and then back to dark. However, the pixel points in the range of 1976–1980 consist of too few pixels. Given that there is a threshold number defined for the gradient zero values of the semi-annular spot, this particular range fails to meet that requirement. Consequently, the gradient change within the pixel points of 1976–1980 is regarded as that of a diffracted spot rather than a characteristic gradient change of the semi-annular spot. At this juncture, the locations within the ranges of 1730–1745 and 1915–1961, as they relate to the original image, are depicted in Figure 11a. It is evident from this illustration that the target spot is captured in a relatively more comprehensive manner within the 939th row of pixels. Through the method of iteratively computing each row of pixels in the image, starting from the top and moving downwards, the end result of the image after the process of de-diffraction is presented in Figure 11b.

After the algorithm processing, it is clearly observable that a significant portion of the diffracted light has been effectively suppressed. In particular, at the focus position, which is the area with the highest light intensity, the elimination of diffracted light is the most pronounced. Moreover, the diffraction artefacts situated between the main spots have been removed to a great extent, enabling a clear observation of the main outline of the spots. The edge position of the semi-annular spot exhibits a relatively weak light intensity, resulting in a less distinct contrast with the background. Consequently, a small number of pixels adjacent to the background may be misidentified as part of the background, causing these pixels to appear black. However, since this phenomenon does not have a substantial impact on the overall resolution of the image, there is no need for further in-depth investigation or correction of this minor issue.

**Figure 12:**
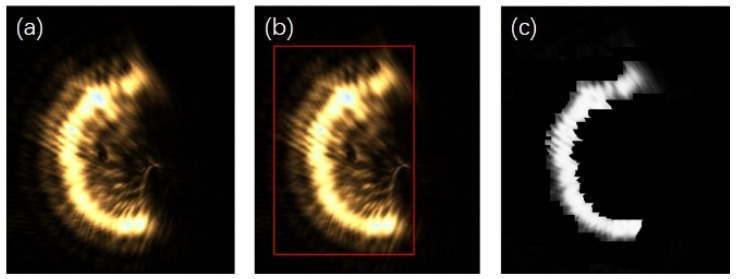
Original and processed images of the spot under strong diffraction. (**a**) is the original image of the spot with strong diffraction effects. (**b**) is the recognition map of the spot localisation interval algorithm for strong diffraction effects. (**c**) is the de-diffracted spot map with strong diffraction effects.

For a single semi-annular spot, when the diffraction interference is strong as shown in Figure 3, the original image with the corresponding removal effect is shown in Figure 12.

As can be discerned from Figure 12a, when the diffraction effect reaches an excessive level, the diffracted spot adheres closely to the semi-annular spot. This intimate juxtaposition renders it arduous to fully separate the diffracted spot from the semi-annular spot. In addition, due to the subpar imaging quality, the semi-annular does not exhibit a regular shape. The boundary between the diffracted light and the main spot is marred by numerous irregular protrusions, or burrs. This leads to the inner and outer diameter edges of the semi-annulus lacking smoothness. As a consequence, the overall imaging quality is significantly diminished. Since errors such as overexposure and improper mounting have the potential to cause the semi-annular spot to deviate from its ideal shape, additional refinement was carried out in Figure 12c with the aim of maximising the improvement in the microscope’s interlayer resolution.

**Figure 13:**
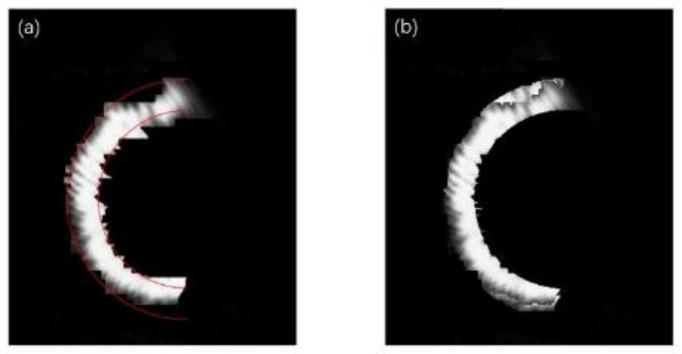
Spot image after de-diffraction and beautified image. (**a**) is optimised on the basis of the original image of the spot after de-diffraction, using the red line as a benchmark. (**b**) is an optimised image.

When the reflective surface of the sample approaches or recedes from the focal plane of the microscope objective, the center of mass of the semi-annular spot will, correspondingly, move towards or away from the center of the image. By closely observing and analysing the movement trajectory of the center of mass of the semi-annular spot, it is possible to make a fairly accurate prediction of the established position of the semi-annular spot. Moreover, the width of the spot provides valuable information that can be used to predict the dimensions of the inner and outer diameters of the semi-annular spot. Leveraging these predictive capabilities, for a distorted semi-annular spot, it becomes feasible to correct it based on the knowledge and patterns learned from the normal movement and characteristics of the spot. The damaged or distorted light spots are refined using the red standard line in Figure 13a as a reference. For the portions of the spot that extend beyond what is considered normal or within the expected boundaries defined by the standard line, these excessive parts are adjusted to match the characteristics of the background. In other words, they are made to blend in with the background, reducing any irregularities or protrusions that deviate from the ideal spot shape. Conversely, for the areas where the spot is lacking or has missing parts, these voids are filled in. The brightness of the pixels used to fill these gaps is determined by the brightness levels of the surrounding pixels in the vicinity of the vacant area. This approach helps to maintain a certain level of consistency and continuity in the spot’s appearance, making it more similar to what an undamaged spot would look like. The outcome of this optimisation process is clearly demonstrated in Figure 13b.

**Figure 14:**
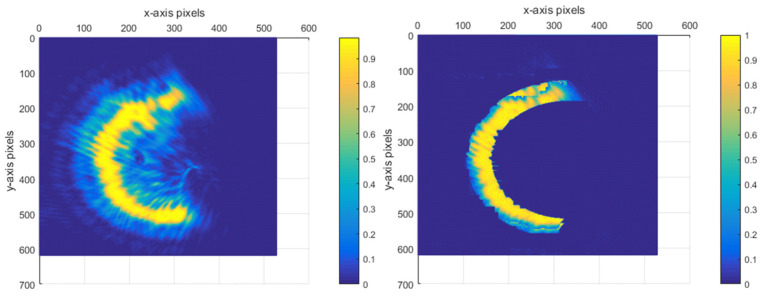
Normalised energy distribution plots for Figure 12a and Figure 13b.

Following the aforementioned treatment, in Figure 13b, the number of pixel points that the ring width occupies is approximately a quarter of the ring width distance in Figure 12a. This significant reduction in the pixel point occupation for the ring width implies a notable improvement in the system’s performance. Specifically, it enables the enhancement of the resolving distance between the current reflective surface and the adjacent out-of-focus reflective surfaces. In fact, this resolving distance has been improved to a quarter of the original limiting resolving distance. The normalised energy diagram depicted in Figure 14 provides a visual representation of the energy distribution and other relevant characteristics of the system after the treatment.

**Figure 15:**
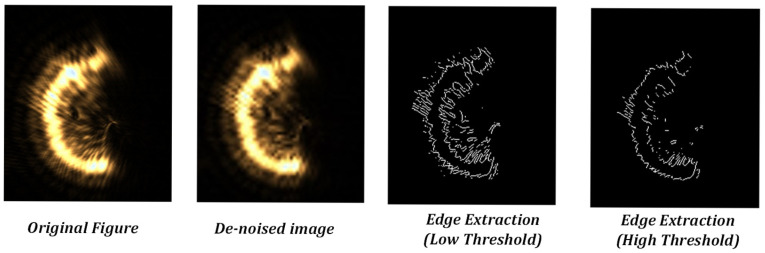
Other optimisation algorithms for speckle map image processing under strong diffraction.

For this semi-annular spot, other algorithms processed as shown in Figure 15 cannot effectively remove the diffraction spot.

**Figure 16:**
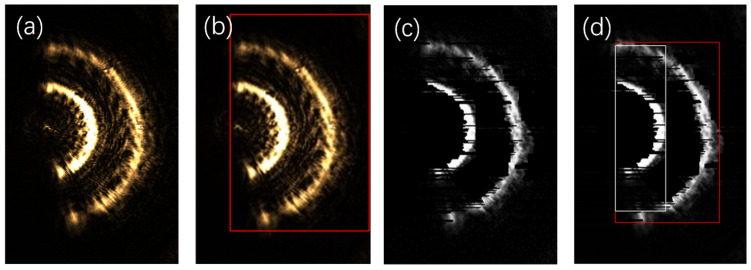
Original and processed images of multiple spots under strong diffraction. (**a**) is the original multi-spot imaging image under strong diffraction. (**b**) is the result after applying the spot recognition algorithm to the original image. (**c**) is the effect of de-diffracting the original image. (**d**) is the result after applying the spot recognition algorithm to the de-diffracting image.

When there are multiple spots in the CCD image under a strong diffraction effect, moving the carrier stage causes all the spots to change in a linear manner simultaneously. To prevent the occurrence of an incorrect relative movement among the spots that could lead to a failure in the focusing process, dynamic capturing is carried out. By using the central pixel of the semi-annular spot as a reference point, it is observed that, as the distance between the carrier stage and the focal plane of the objective lens varies, the center of this pixel undergoes a linear translation. This linear relationship provides a basis for predicting the shape of the spot. Based on the result of this pre-judgment, real-time corrections can be made to the sample spot that has been incorrectly imaged by the CCD. Take Figure 16a as an example.

The sample is equipped with three reflective surfaces. As the carrier stage gradually moves closer to the objective lens, when the first reflective surface reaches the focal position of the objective lens, the spot corresponding to the first reflective surface will converge into a single point. Meanwhile, the spots of the other two reflective surfaces, due to being out of focus, will take on the form of semi-annular spots. Nonetheless, the presence of aberration causes an abnormality in the imaging spots of the reflective surfaces. It is evident from Figure 16a that not only is the brightness of the spot at the focal point extremely low, but the shape of the spot has also been distorted. Moreover, because the rings are positioned closely together and the diffracted spot exhibits high brightness, the AF system made an incorrect judgment, believing that there was only a single spot present at that moment, as illustrated in Figure 16b. To rectify the error of the autofocus system, a crucial first step was to carry out a de-diffraction operation on the image in Figure 16a. The outcome of this operation is depicted in Figure 16c. Following the de-diffraction process, when the calibration was performed again, the autofocus system was then able to accurately recognise the two out-of-focus semi-annular spots. As shown in Figure 16d, these spots were clearly marked with white and red boxes, indicating that the system had now correctly identified their presence and positions.

**Figure 17:**
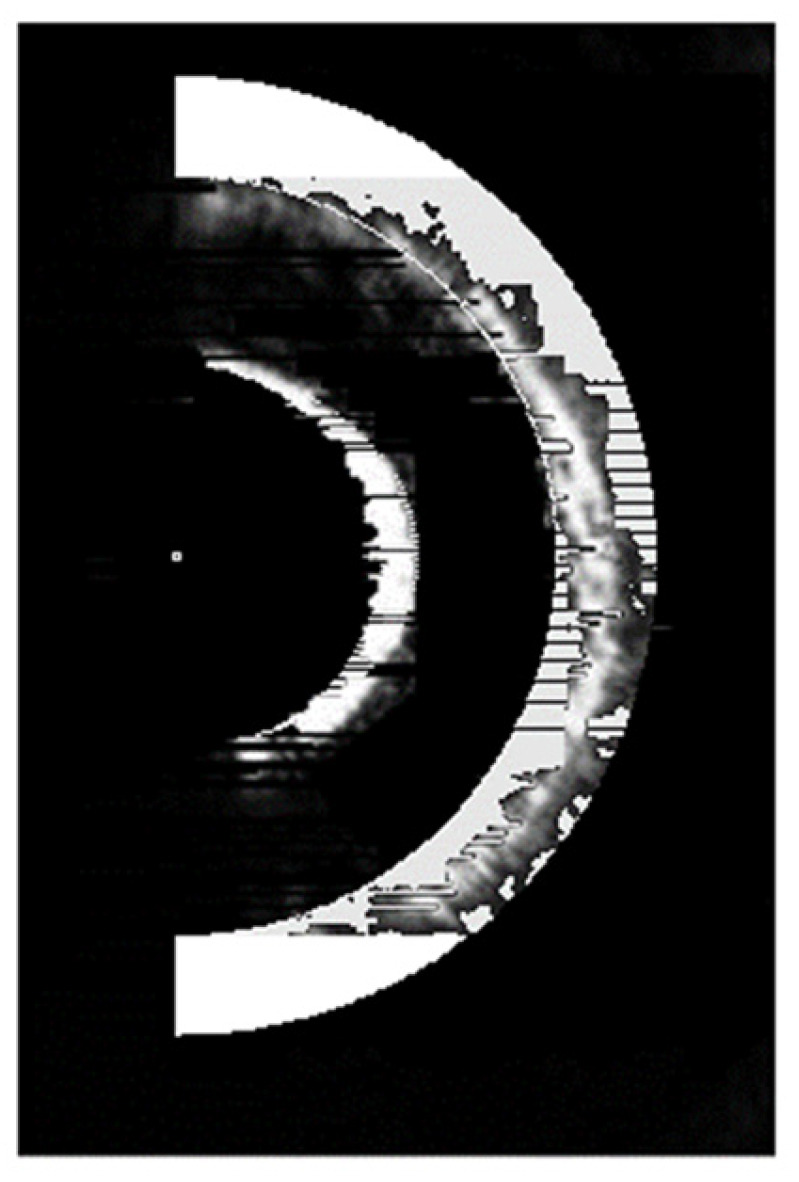
Final image after correction of the spot imaging image under strong diffraction.

Since the spot information of a reflective surface is missing in the image, in order to fill in the missing annular spot, the starting position of the inner diameter of the missing spot can be calculated by Equation (2) based on the position of the inner diameter of the neighbouring semi-annular spot in the missing part of the image. In Figure 16d, the width of the first semi-annular is 17 pixels, and the width of the second semi-annular is 25 pixels. Given that the spacing between each layer of the reflective surface of the experimental sample is the same, as can be deduced from Equation (1), at this moment, the width of the second half-ring should be twice that of the first half-ring, namely, 34 pixels, which is more than 10 pixels greater than the actual measured width. Likewise, the coordinates of the center of the inner diameter of the first semi-annular are (118, 180), and the coordinates of the center of the inner diameter of the second semi-annular are (182, 180). Therefore, according to Equation (2), the coordinates of the center of the spot of the focusing reflective surface can be calculated to be (54, 180). Based on the above analysis, Figure 16d was corrected. This involved filling in the missing spot and rectifying the ring width. Under these experimental conditions, the theoretical resolving distance of the Airy spot is 0.68 μm, which approximately occupies 2 pixels. Hence, the focus was supplemented with an approximate circle having a value corresponding to 2 pixels, as presented in Figure 17.

**Figure 18:**
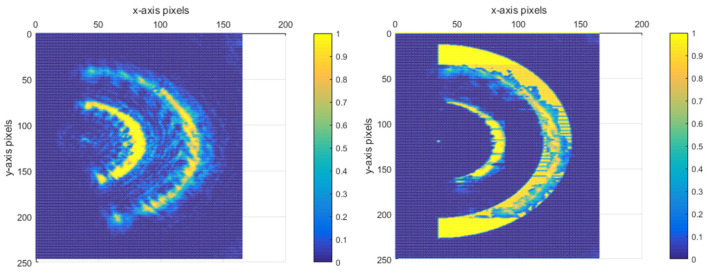
Normalised energy distribution plots for Figure 16a and Figure 17.

As is evident from Figure 16b, initially, there were two semi-annular spots that were extremely close to each other, surpassing the resolution limit distance. However, following the image processing while maintaining their relative distance unchanged, not only could these two spots be resolved distinctly, but the distance between them could also be further reduced. In fact, the distance could be shortened to 0.23 times of the original distance, which is approximately a quarter of the initial value. This remarkable outcome indicates that, through the anti-diffraction processing of the algorithm, the interlayer resolution of the semi-annular spots has been significantly enhanced. Specifically, it has been increased to a quarter of the original limiting resolution. The normalised energy diagram corresponding to this improved state is illustrated in Figure 18.

## 5. Conclusions

This paper presents a new algorithm to address the resolution degradation in a multilayer autofocus microscope caused by diffraction effects. It reduces the amount of image processing operations through the interval detection of imaging spots on the CCD. A gradient iteration algorithm is used to remove diffraction for spots within the interval, and reduction fitting is carried out for low-quality imaging spots. Experimental results show that the algorithm effectively removes diffracted spots near the main spot, decreasing the limiting distance between original semi-annular spots to a quarter of the initial value and enhancing the interlayer resolution of the sample’s reflective surfaces. Therefore, this algorithm has an important role in biogenetic sequencing, wafer detection, etc.

## Data Availability

The data are contained within the article.
